# Distrust and expertise: Can scientific journals continue as gatekeepers?

**DOI:** 10.1098/rsnr.2016.0031

**Published:** 2016-08-31

**Authors:** Vanessa Heggie

**Affiliations:** Social Studies in Medicine, College of Medical and Dental Sciences, University of Birmingham, Edgbaston, Birmingham B15 2TT, UK

When considering both the past and the future of the scientific journal, one key feature of this form of publication that needs to be kept at the centre of our analysis is expertise. Amidst the calls to democratize access to research, to broaden participation, and to create a more inclusive and accessible scientific practice for the twenty-first century, it is easy to find the notion of expertise a difficult one to include in the new landscape of publication. It is easy to imagine the drive to author-pays open access as a form of direct access to expertise, enabling anyone to read the original papers and in some cases see the data generated by scientists and other researchers. But at the same time, this model acts to erase the reality and significance of expertise, by assuming that access to research is the same as access to the understanding or meaning of research—by prioritizing the individual need for direct access to a consumable material, and by figuring all forms of gatekeeping as essentially anti-egalitarian, exclusivist, and negative. This is an extraordinarily naive understanding of how research ideas reach a public audience, fostered by a failure to recognize the diversity of the ‘publics’ that exist as potential readers of scientific journals. It is not a mistake made by successful science journals, past or present. Nineteenth-century editors were acutely aware of the variety of reading audiences available to them, and in an era of for-profit science publishing, they perhaps had a more acute ear for the levels of expertise of their audiences, and, crucially, an understanding of the many reasons why someone might want to read a scientific publication. These audiences might range from the researcher with high expertise but low institutional access to libraries with comprehensive journal subscriptions, to the journalist with medium expertise and a need to translate research for a lay audience, to the lay audience themselves.

This erasure of expertise, and distrust of ‘gatekeepers’, has recent historical precedent. The long, detailed descriptions of counter-indications and side-effects available in packets of medication are a consequence of concerns (particularly relating to the contraceptive pill) in the 1970s that doctors were not acting as efficient gatekeepers, and were not providing patients (in this case, specifically women) with enough information to be fully informed consumers of medicine. Yet, amongst those concerned with scientific literacy today, there is genuine anxiety about the ability of many of us to estimate and understand risk; so there is a serious question here about whether these leaflets achieve what they were intended to—that is, to produce better informed patient-consumers. Allowing ‘data to be free’ does not seem to remove the need for expert communicators and adjudicators. Realistically, journals are therefore likely to retain at least one role: curating and guaranteeing expertise. Even where an individual cannot understand the contents of a paper, the name of the journal may still instil confidence. In a crowded information marketplace, journals can be used as a useful shorthand to sift and rank masses of—often unintelligible—data. The future role of the journal seems very similar to its past role, despite changes in format and funding: to disseminate research, to provide authority. Prioritizing one half of this role and ignoring the other will lead to poor solutions to the problems journals face; and critics who ignore the variable expertise and interests of the people to whom one might ‘disseminate’ are probably the biggest threat to a stable future for scientific journals.

